# Design, growth, and characterization of Y_2_Mo_4_O_15_ crystals for Raman laser applications†

**DOI:** 10.1039/d0ra08609f

**Published:** 2021-01-04

**Authors:** Xiangmei Wang, Zeliang Gao, Chunyan Wang, Xiaojie Guo, Youxuan Sun, Yu Jia, Xutang Tao

**Affiliations:** State Key Laboratory of Crystal Materials, Shandong University Jinan 250100 China txt@sdu.edu.cn gaozeliang@sdu.edu.cn; Key Laboratory for Special Functional Materials of Ministry of Education, School of Materials Science and Engineering, Henan University Kaifeng Henan 475001 China; International Laboratory for Quantum Functional Materials of Henan, School of Physics and Microelectronics, Zhengzhou University Zhengzhou 450001 China

## Abstract

A new crystal Y_2_Mo_4_O_15_ with dimensions of 14 × 12 × 5 mm^3^ was successfully grown *via* a top-seeded solution growth (TSSG) method. The crystal structure shows that Y_2_Mo_4_O_15_ crystallizes in the monoclinic space group *P*2_1_/*c* (No. 14, *a* = 6.8110(4) Å, *b* = 9.5833(6) Å, *c* = 10.5124(7) Å, *β* = 105.512(7)°, and *Z* = 2) with Mo_4_O_15_ and YO_7_ polyhedra as basic structural units. Optical transmittance spectra of the Y_2_Mo_4_O_15_ crystal exhibited a broad transmission range from 345 nm to 5575 nm. The group theory calculation and spontaneous Raman spectra show that the Y_2_Mo_4_O_15_ crystal has 63 IR-active modes (32A_u_ + 31B_u_) and 60 Raman-active modes (30A_g_ + 30B_g_). The strongest Raman shift is located at 953 cm^−1^ caused by the asymmetric stretching vibrations of the Mo–O bonds. The spontaneous Raman spectra and the possibility of rare-earth doping to the Y_2_Mo_4_O_15_ crystal indicate the Y_2_Mo_4_O_15_ crystal to be a promising Raman and self-Raman crystal.

## Abstract 2

This work reports the crystal structure and physical properties of the Y_2_Mo_4_O_15_ crystal and its potential use in the Raman laser.

## Graphical abstract

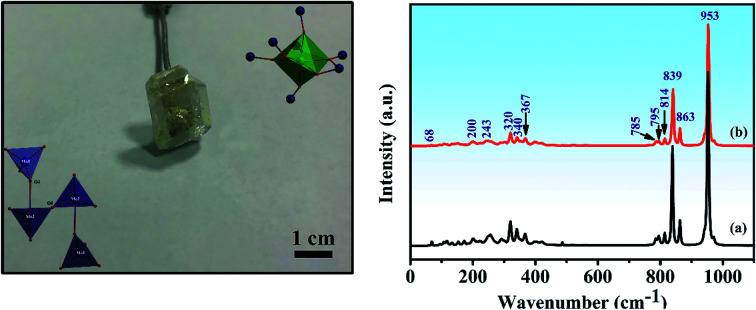

## Introduction

Stimulated Raman Scattering (SRS) provides an efficient and simple method for broad laser sources. A lot of crystals such as diamond,^1^ LiIO_3_,^2^ Ba(NO_3_)_2_,^3^ and YVO_4_ (ref. 4) have been studied for Raman laser applications. In order to simplify the laser configuration, the self-Raman laser for rare-earth doped crystals, including Nd:YVO_4_,^5^ Nd:GdVO_4_,^6^ and Yb:KGd(WO_4_)_2_,^7^ have been applied as laser and SRS crystals simultaneously. Recently, both the molybdate and tungstate, such as CaMoO_4_,^8^ SrMoO_4_,^9^ BaTeMo_2_O_9_,^10,11^ BaWO_4_,^12^ SrWO_4_,^13^ and CaWO_4_,^8^ have been paid attention owing to their excellent SRS properties. Although the above molybdate and tungstate crystals exhibit excellent Raman property, they are difficult for rare-earth doping due to the mismatch of their cation valence. It is well-known that the Raman spectra are mainly determined by anionic groups. The Raman shifts located at 872–930 cm^−1^, which are valuable for Raman laser, are due to the Mo–O vibrations. Therefore, molybdate crystals with trivalent cation possess good Raman properties, making them very promising hosts of the active dopants for self-Raman materials.

Y_2_Mo_4_O_15_ belongs to the monoclinic system in the space group *P*2_1_/*c* with *a* = 6.8185(2) Å, *b* = 9.5913(3) Å, *c* = 10.5299(3) Å, *β* = 105.586(2)°, and *Z* = 2.^14^ The Y_2_Mo_4_O_15_ and rare-earth doped Y_2_Mo_4_O_15_ powders have been synthesized, and special attention is paid to the investigation of these systems, which are used as luminescent host materials for white LEDs.^15–19^ It is well known that the Y sites are easily accessible for various rare-earth ion substitutions. Therefore, it allows the production of active solid state self-Raman lasers with effective doping of numerous rare-earth ions.

In this study, for the first time, the Y_2_Mo_4_O_15_ crystal was grown by the top seed solution growth method. The crystal structure, optical properties, and Raman spectra have been studied in detail. The lattice vibrations of the Y_2_Mo_4_O_15_ crystal were analysed by the space group theory. The experimental results and theoretical calculations indicate that the Y_2_Mo_4_O_15_ crystal is a candidate for Raman and self-Raman crystals.

## Experimental

### Synthesis

Y_2_Mo_4_O_15_ powders were synthesized by a high temperature solid-state reaction method. The stoichiometric amounts of Y_2_O_3_ (99.99%, Alfa-Aesar) and MoO_3_ (99.5%, Alfa-Aesar) were thoroughly mixed in an agate mortar and then pressed into a pellet. The pellet was transferred into a platinum crucible in a programmable muffle furnace. After that, the column was preheated to 650 °C at a rate of 10 °C min^−1^ and then heated at a rate of 2 °C min^−1^ from 650 °C to 860 °C gradually with a holding time of 72 h. Several intermittent re-grindings were performed until the pure powders were obtained.

### Powder X-ray diffraction (PXRD)

An automated Bruker D8 ADVANCE X-ray diffractometer equipped with a diffracted monochromator set for Cu Kα (*λ* = 1.5418 Å) radiation was used to measure the XRD patterns. The measurements were performed in the angular range (2*θ*) from 10° to 90°, with a scanning step width of 0.02° and a scanning speed of 20° min^−1^.

### Thermal stability

A series of thermogravimetric and differential scanning calorimetry (TG-DSC) had been done on ground Y_2_Mo_4_O_15_ polycrystals in a shielded nitrogen environment using a TGA/DSC1/1600HT (Mettler Toledo Instruments).

### Single-crystal growth

After several attempts, some Y_2_Mo_4_O_15_ crystal seeds were obtained for the first time by a spontaneous nucleation method using Li_2_CO_3_–MoO_3_ as a flux. In the crystallization region, a reaction mixture of Li_2_CO_3_, MoO_3_, and Y_2_O_3_ at the molar ratio of 1 : 8 : 2 was adequately ground and then placed in a platinum crucible. The reaction mixture was subsequently heated to 960 °C and maintained at that temperature for 96 h to guarantee an entirely homogeneous solution. A platinum wire was dipped into the surface of the solution at 960 °C, and then at a rate of 5 °C h^−1^, the temperature was decreased to 776 °C to obtain the millimetre-sized crystals by spontaneous nucleation. A single crystal of Y_2_Mo_4_O_15_ was obtained *via* the top seeded solution growth (TSSG) technique. In addition, the saturation temperature of the solution was obtained by the testing seed crystal method. A regular and transparent crystal seed was gradually brought into the solution at 2 °C above the saturation temperature, and was maintained for an hour to melt slightly surface impurities. After that, the temperature was rapidly reduced to the saturation temperature, and then a fairly slow cooling program of approximately 0.02 °C d^−1^ was adopted to carry out the growth procedure. When the sizable Y_2_Mo_4_O_15_ crystal was harvested, the furnace temperature was cooled at a rate ranging from 30 to 50 °C h^−1^.

### Single-crystal X-ray diffraction

Crystallographic data were collected on a Bruker AXS SMART diffractometer with monochromatic Mo K*α* radiation (*λ* = 0.71073 Å) at room temperature.^20^ The data integration and unit cell refinement were determined by the INTEGRATE program of the standard APEX II software, and absorption corrections were performed with the SCALE program for the area detector.^20^ The structure was solved directly by Fourier synthesis and then refined according to the full-matrix least square techniques fitting on *F*_0_^2^ using the SHELXTL program.^21^ The details of the crystal structure (CCDC 2027158) can be obtained.

### Laue back-reflection measurement

The back-reflection Laue technique is widely used for orientation and crystallinity assessment of a single crystal.^22^ A crystal facet (100) slice of Y_2_Mo_4_O_15_, mechanically polished on both sides, whose dimensions are 4 × 4 × 1 mm^3^, was employed for the Laue diffraction measurement to assess the crystalline perfection by a real time back-reflection Laue camera system (Multiwire MWL 120 with Northstar software).

### UV-visible diffuse reflectance spectra

UV-vis diffuse reflectance measurements of Y_2_Mo_4_O_15_ were performed on a Shimadzu UV 2550 spectrophotometer equipped with an integrating sphere, and the baseline correction was performed using a calibrated reference sample of powdered barium sulfate (BaSO_4_) over the wavelength range extending from 200 to 800 nm. The band-gap energy of Y_2_Mo_4_O_15_ was calculated according to the Tauc expression.^23^ A thin {100} crystal plate (4 × 4 × 1 mm^3^) was cut from the as-grown Y_2_Mo_4_O_15_ crystal. The optical transmittance spectrum in the visible and near IR range (0.2–2 μm) was collected on a Hitachi U-3500 UV-vis-IR spectrometer, and the spectra in the mid-IR (2–6 μm) region were recorded on a Nicolet NEXUS 670 FTIR spectrometer.

### X-ray photoelectron spectra

X-ray photoelectron spectra (XPS) were recorded using a Thermo Fisher ESCALAB 250 X-ray photoelectron spectrometer with monochromatic Al Kα X-ray radiation. The binding energy of the samples was calibrated using the C 1s peak (284.6 eV) as a reference. The spectra were successfully deconvoluted using a Gaussian–Lorentzian curve fitting program after background subtraction.

### Spontaneous Raman spectroscopy

The spontaneous Raman spectra of Y_2_Mo_4_O_15_ were recorded at room temperature using 1064 nm radiation as the pump source using a Jobin Yvon T64000 spectrometer with dimensions of 4 mm × 4 mm × 1 mm^3^ square slice as a sample. Raman spectra with 2 cm^−1^ spectral resolution in the frequency range of 0 to 1100 cm^−1^ were collected.

### Computational details

*Ab initio* density functional theory calculations were performed using the Vienna *Ab initio* simulation package (VASP) code.^24^ The Perdew–Burke–Ernzerhof (PBE) function^25^ was employed to calculate the exchange-correlation potential under the generalized gradient approximation (GGA).^26^ A kinetic energy cut-off of 500 eV was used for the plane wave basis set. The Brillouin zone sampling was carried out with a 7 × 7 × 5 Monkhorst–Pack grid. Convergence criteria employed for both the electronic self-consistent relaxation and ionic relaxation are set to be 10^−8^ and 10^−4^ eV Å^−1^ for energy and force, respectively. Gaussian broadening was implemented with a smearing width of 0.05 eV. The calculations of the phonon spectrum and phonon density of states were performed using a 2 × 2 × 1 supercell in the framework of the density functional perturbation theory (DFPT).^27^ Force-constants supercell has been obtained using the VASP code. The PHONOPY code^28^ has been used to calculate the phonon frequencies and phonon density of states.

## Results and discussion

### Synthesis and characterization of polycrystalline

#### Y_2_Mo_4_O_15_

**Fig. 1:**
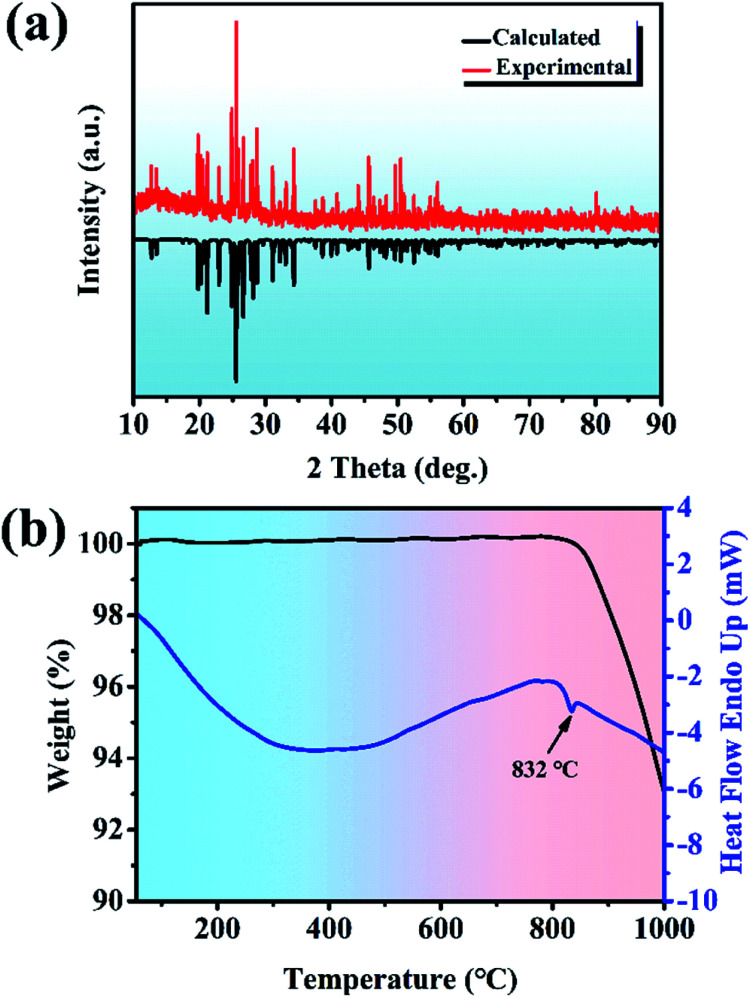
(a) Experimental PXRD patterns for polycrystalline Y_2_Mo_4_O_15_ (pure-phase) and calculated PXRD patterns for polycrystalline Y_2_Mo_4_O_15_. (b) DSC and TGA curves for polycrystalline Y_2_Mo_4_O_15_.

The Y_2_Mo_4_O_15_ polycrystalline materials were synthesized by the traditional solid-state reaction. XRD patterns of Y_2_Mo_4_O_15_ are depicted in Fig. 1a, together with the reference pattern of Y_2_Mo_4_O_15_ for comparison, which indicates that the as-prepared samples are in the pure phase. DSC and TGA measurements were carried out to measure the thermal stability of the Y_2_Mo_4_O_15_ crystal (shown in Fig. 1b). There is a single evident endothermic peak at 832 °C. It indicates that Y_2_Mo_4_O_15_ potentially decomposes before its melting point. The PXRD detection of the solid residue after heating at 900 °C for an hour revealed that Y_2_Mo_4_O_15_ decomposed to YMoO_4_. This evidence indicates that Y_2_Mo_4_O_15_ is an incongruent melting compound. Accordingly, large-sized Y_2_Mo_4_O_15_ crystals should be grown *via* a high-temperature flux method.

### Single-crystal growth

**Fig. 2:**
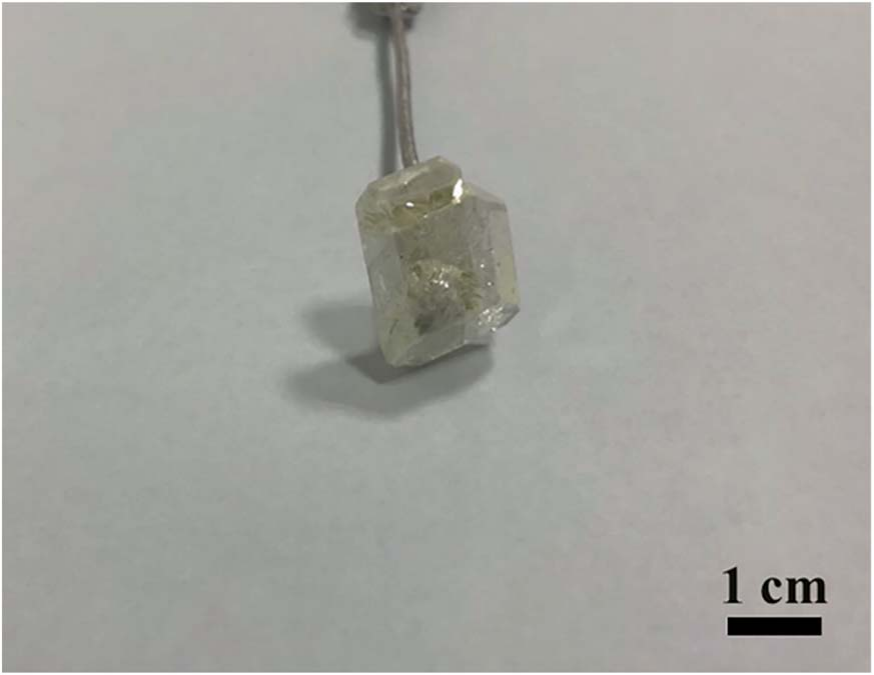
Photograph of the as-grown Y_2_Mo_4_O_15_ crystal.

Flux growth is worthwhile to pursue because it readily allows the crystal growth at a temperature below the melting point. In addition, the crystal grown from flux has a regular morphology and low dislocation density. Therefore, the single crystal Y_2_Mo_4_O_15_ was grown with the TSSG method. The selection of the flux system is extremely crucial to the growth of high-quality crystals. MoO_3_ with a low melting point (793 °C) and outstanding solubility has been widely used as a self-fluxing agent to grow new oxide crystals.^29^ Moreover, a special effect of Li^+^ cations potentially makes the grown crystal surface more visible.^30^ Using the Li_2_CO_3_–MoO_3_ flux (molar ratio, Li_2_CO_3_ : MoO_3_ = 1 : 8), Y_2_Mo_4_O_15_ was prone to crystallization. Taking advantage of the seed crystals with suitable size and quality obtained by spontaneous crystallization, a colorless and transparent single Y_2_Mo_4_O_15_ crystal with a size of 14 × 12 × 5 mm^3^ was successfully grown by the TSSG method (see Fig. 2). More importantly, this method is available to achieve the crystal growth at a temperature much lower than its decomposition point.^31^

### Crystal structure of Y_2_Mo_4_O_15_

**Fig. 3:**
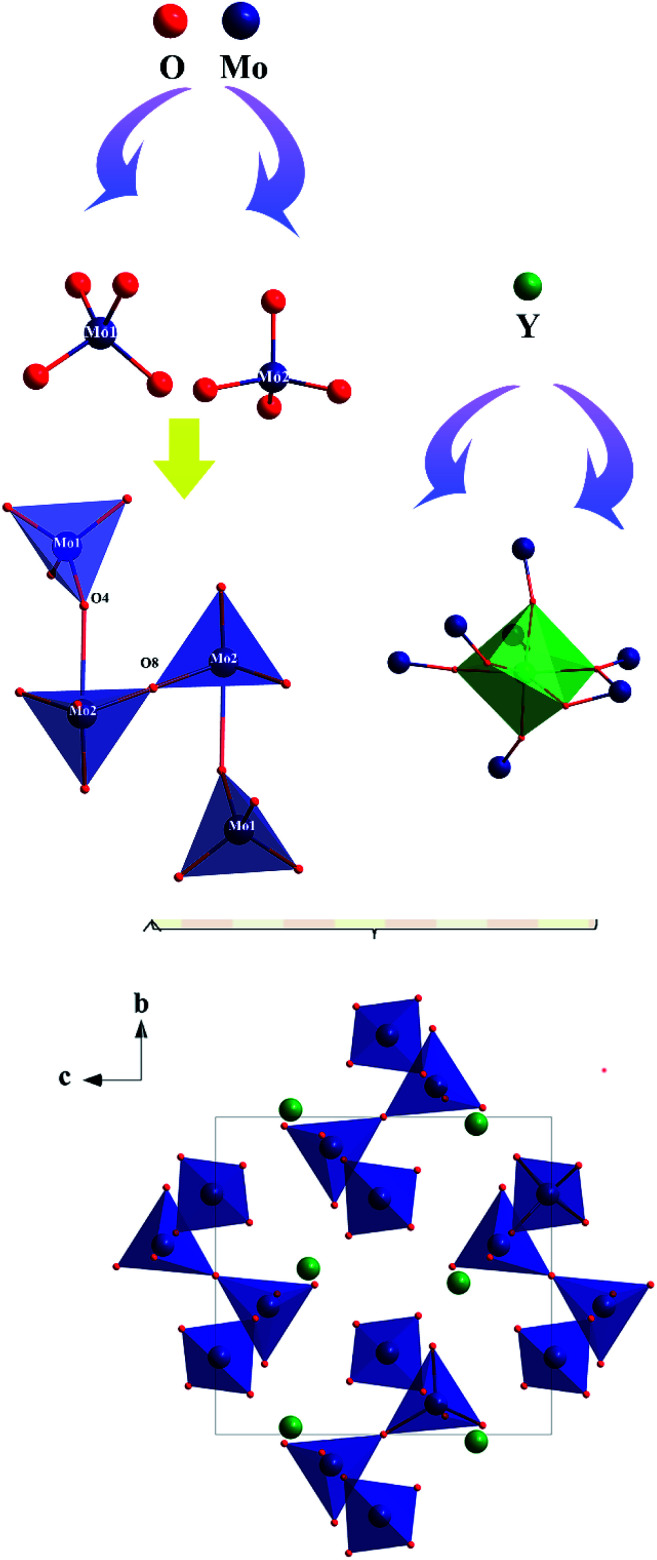
Ball-and-stick diagram of the Y_2_Mo_4_O_15_ crystal structure.

The preliminary crystallographic information on a polycrystalline sample of Y_2_Mo_4_O_15_ is reported. To provide better insights into the crystal structure, single crystal X-ray diffraction data of Y_2_Mo_4_O_15_ were determined at 293 K in detail. It crystallizes in the monoclinic system with the space group of *P*2_1_/*c* (No. 14). The refined crystallographic data and details of the experimental conditions of Y_2_Mo_4_O_15_ are given in Tables S1–S3 of the ESI.† According to the structure (see Fig. 3), there are two different molybdate units in the present structure. Mo(1) is surrounded by four oxygen atoms to form a fairly regular Mo(1)O_4_ tetrahedron. For Mo(2), except for the four nearest coordinated oxygen atoms, two Mo(2)O_4_ tetrahedra are connected by O(8) to form a Mo(2)_2_O_7_ pyromolybdate, in which the bridging O(8) atom between the two vertex-shared MoO_4_ tetrahedra reside on an inversion center. In addition, Mo(2)_2_O_7_ pyromolybdate is loosely connected to two Mo(1)O_4_ tetrahedra and forms an entire Mo_4_O_15_ unit, as shown in Fig. 3. Furthermore, the anionic environment of the crystallographically distinct Y^3+^ position is coordinated with seven oxygen atoms (CN = 7) to form a singly capped trigonal prism with the Y–O distances ranging from 0.247 nm to 0.355 nm (see Table S2†). The crystal structure is consistent with the result reported by Laufer *et al.*^14^ Due to the flexibility of coordination geometry for the Y^3+^ center,^32^ the Y_2_Mo_4_O_15_ compounds are isomorphous with the previously reported Ln_2_Mo_4_O_15_ (Ln = Y, Dy, Ho, and Tm),^33^ which means doping with rare-earth ions is easy for the Y^3+^ position. In addition, MoO_4_ tetrahedron and YO_7_ polyhedron active vibrations laid the theoretical foundation for Raman scattering.

### Laue back-reflection patterns

**Fig. 4:**
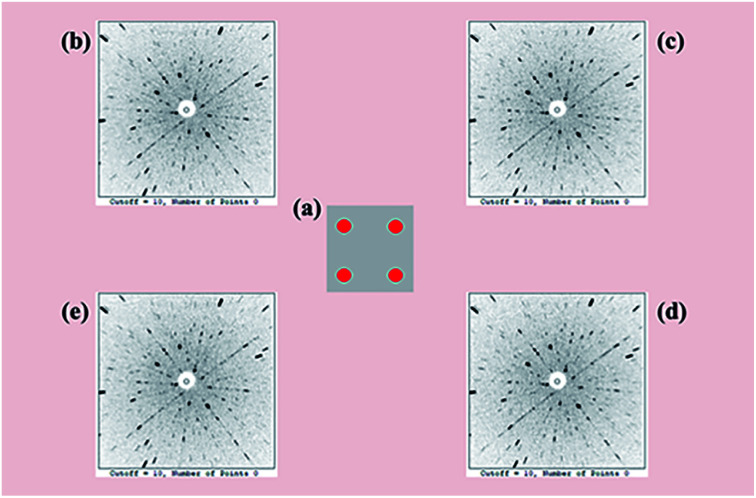
(a) Schematic positions (red dots) of the Laue back-reflection measurement on the crystal piece. From clockwise rotation (b–e), characteristic Laue back-reflection patterns at different positions with the X-ray beam hitting the crystal piece.

To facilitate a precise investigation of the crystallinity of an as-grown crystal, the Laue back-reflection measurement is broadly exploited.^34^ The characteristic Laue back-reflection patterns of different positions in the (100) crystal wafer are accordant and distinct, as shown in Fig. 4. It demonstrates that the crystal quality of the Y_2_Mo_4_O_15_ crystal is high enough, which furnishes the basis for performing the measurements and evaluations of intrinsic physical properties.

### Optical properties

**Fig. 5:**
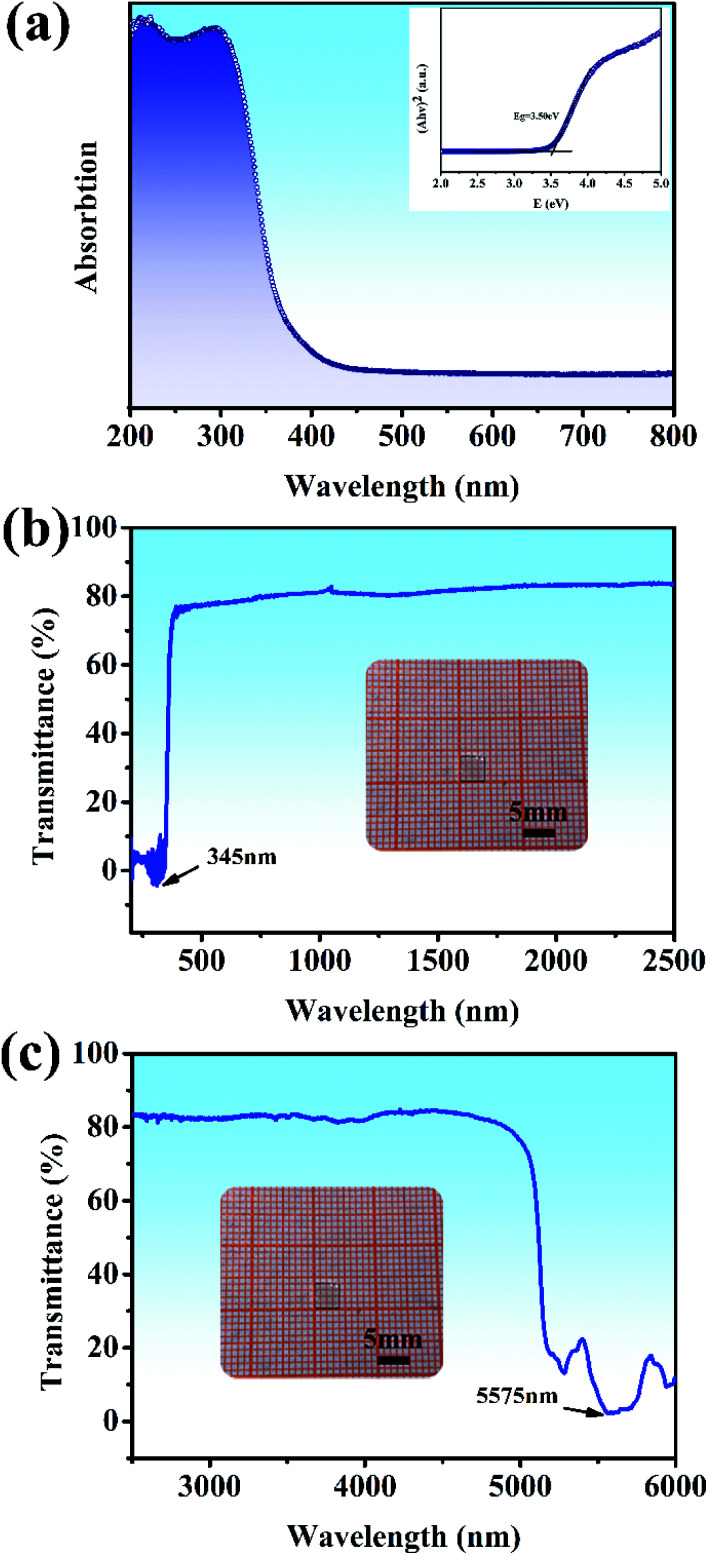
(a) UV-visible diffuse reflectance spectra data for ground powders of the Y_2_Mo_4_O_15_ crystal. The inset shows the relationship between K/S and E (eV); (b) and (c) UV-vis and IR transmission spectra of the Y_2_Mo_4_O_15_ single crystal. Inset: Y_2_Mo_4_O_15_ crystal wafer of dimensions 4 × 4 × 1 mm^3^.

The UV-vis diffuse reflectance spectrum based on the ground powders of the Y_2_Mo_4_O_15_ crystal is illustrated in Fig. 5a. As presented in Fig. 5a (inset), the indirect band gap E_g_ of Y_2_Mo_4_O_15_ is estimated to be about 3.50 eV by fitting the corresponding Tauc plot of the Kubelka–Munk transformed UV-vis diffuse reflection data,^35^ which is similar to the previous work.^16^ The optical transmission spectra (Fig. 5b and c) of the Y_2_Mo_4_O_15_ crystal wafer (Fig. 5b and c, inset) were performed at room temperature. The UV *λ*_cut-off_ of Y_2_Mo_4_O_15_ is located at 345 nm, which is more reliable than that of the powdered Y_2_Mo_4_O_15_ (334 nm).^16^ In the mid-IR range, the Y_2_Mo_4_O_15_ crystal displays high transparency up to 5.1 μm with an absorption edge of 5.575 μm. The Y_2_Mo_4_O_15_ crystal exhibits a wider transparent window than that of ternary molybdate and tungstate, such as SrMoO_4_ (0.31–3 μm), BaWO_4_ (0.26–3.7 μm), and SrWO_4_ (0.3–2.7 μm). These results indicate that Y_2_Mo_4_O_15_ is a promising alternative for a large variety of Raman applications, which can be extended over a wide spectral range.

### Electronic structure

**Fig. 6:**
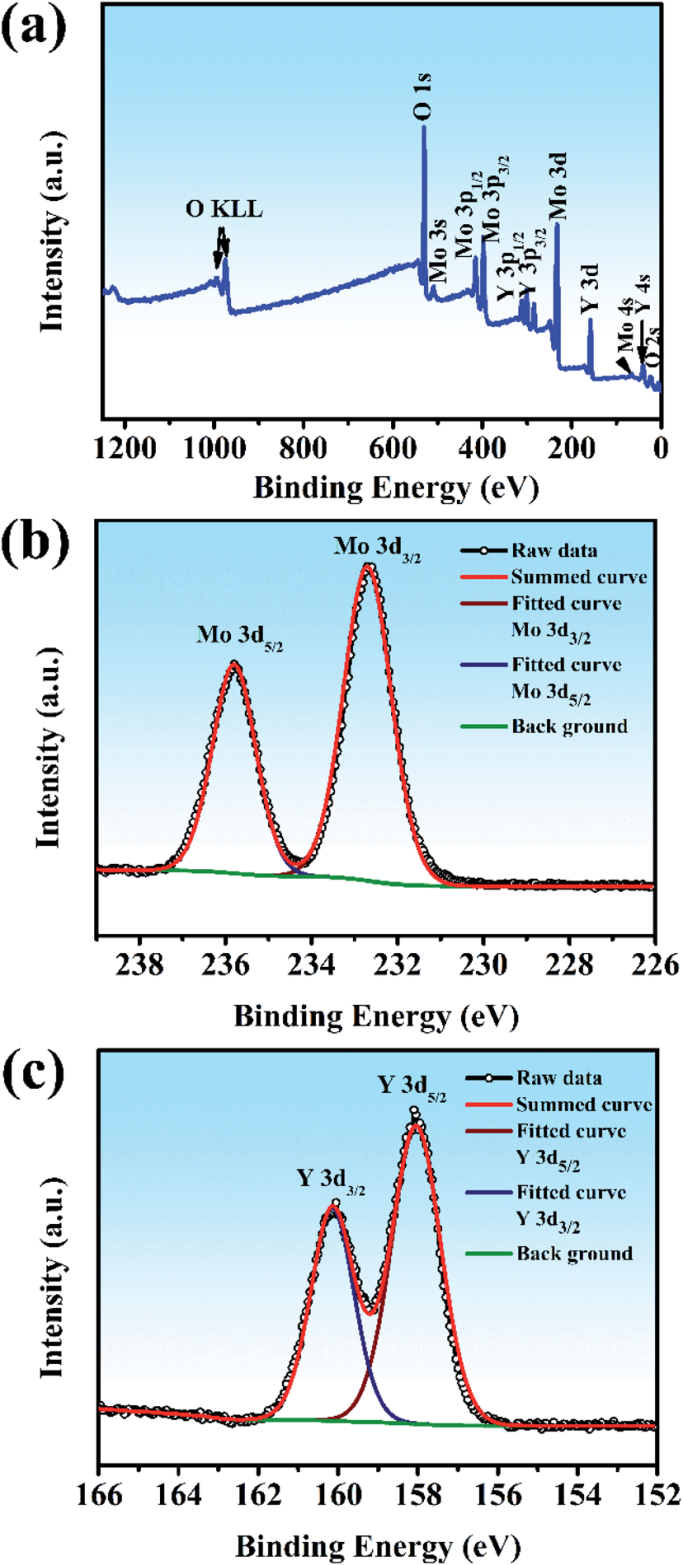
(a) Survey X-ray photoelectron spectra of Y_2_Mo_4_O_15_ crystals. The different elements and their associated electronic states are denoted for respective peaks: high-resolution X-ray photoelectron spectra of (b) Mo 3d core level and (c) Y 3d core level.

XPS was used to shed light on the surface composition and binding interactions of the Y_2_Mo_4_O_15_ crystal (Fig. 6). All the spectral features from the curves, except for the C 1s core level, can be attributed to the core-levels or Auger lines of the constituent elements of Y_2_Mo_4_O_15_. The high-resolution XPS spectra of Mo 3d and Y 3d core-level spectra correspond to the simple spin–orbit doublets. The binding energy values of the Mo 3d_5/2_ (235.8 eV), Mo 3d_3/2_ (232.7 eV) Y 3d_3/2_ (160.15 eV), and Y 3d_5/2_ (158.05 eV) core levels in Y_2_Mo_4_O_15_, as obtained by the present XPS measurement, are similar to the previous reports on Y_2_Mo_4_O_15_,^36^ and correspond to those of Y and Mo in the formal valence states 3+ and 6+. The results show that the Y_2_Mo_4_O_15_ crystal has stable chemical properties.

### Raman spectra

**Fig. 7:**
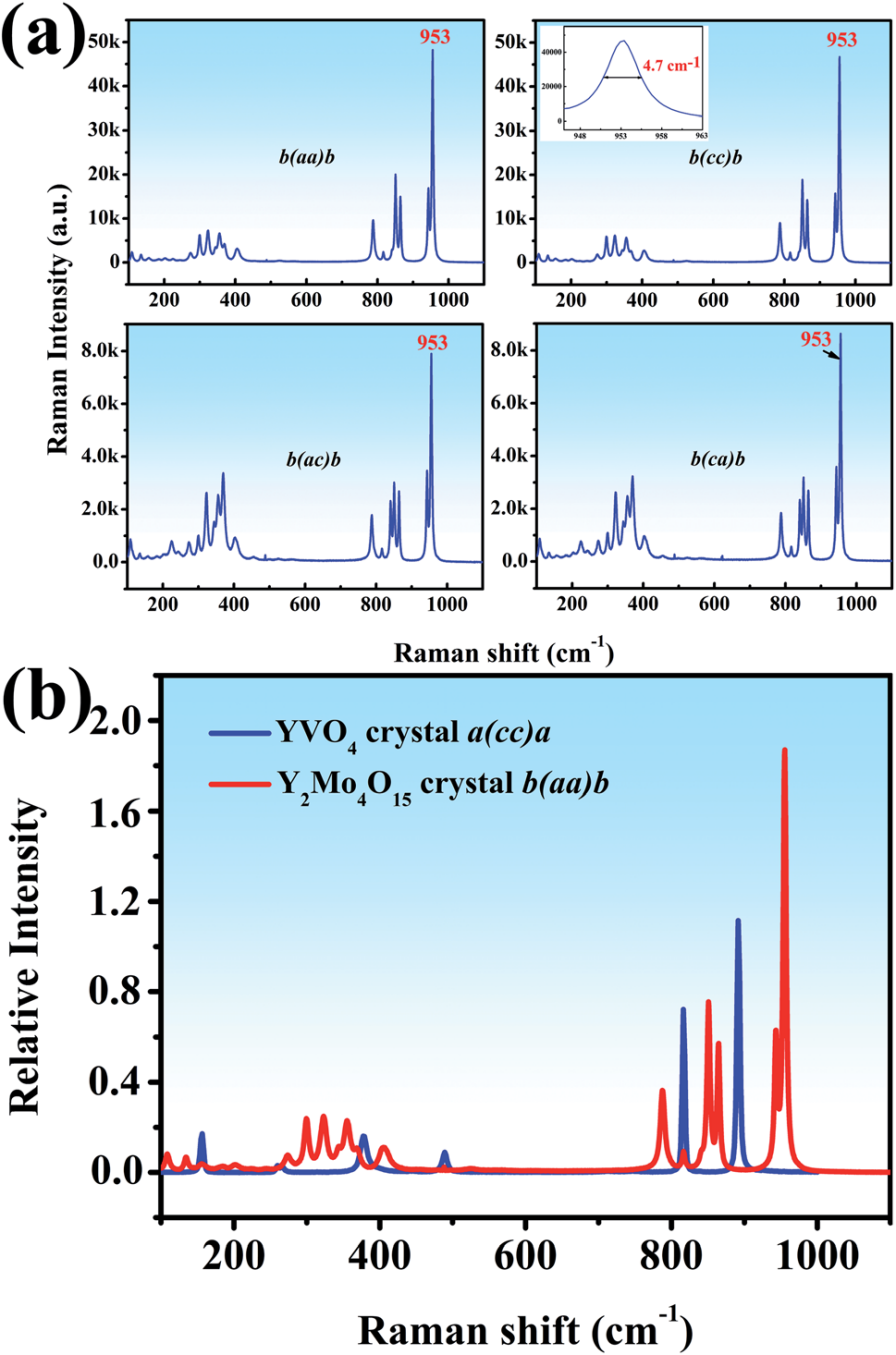
(a) The spontaneous Raman spectra of Y_2_Mo_4_O_15_ along the *b* axis (b) relative spontaneous Raman spectra of YVO_4_ and Y_2_Mo_4_O_15_.

Both the polarized Raman spectrum and spontaneous Raman spectra are beneficial to the analysis of vibrational modes.^37^ We report the spontaneous Raman spectra of the Y_2_Mo_4_O_15_ single crystal along the *b*-axis with different Raman configurations, as shown in Fig. 7a. It can be seen that both *b(aa)b* and *b(cc)b* Raman configurations exhibit the strongest Raman shifts at 953 cm^−1^. It is well known that Raman spectra are mainly determined by anionic groups. As is shown in Fig. 7a (inset), the peak linewidth is about 4.7 cm^−1^, which is shorter than that of CaMoO_4_ (5.5 cm^−1^) but wider than that of BaMoO_4_ (1.85 cm^−1^).^38^

To evaluate the potential Raman property, the Raman shift intensity of Y_2_Mo_4_O_15_ crystal is at 953 cm^−1^ in the *b(aa)b* polarization configuration is compared to that of the YVO_4_ crystal. It is about 1.68 times than that of the Raman shift at 890 cm^−1^ in YVO_4_, which indicates excellent Raman scattering properties (Fig. 7b).

### Raman spectral characteristics of *P*2_1_/*c* symmetry

**Fig. 8:**
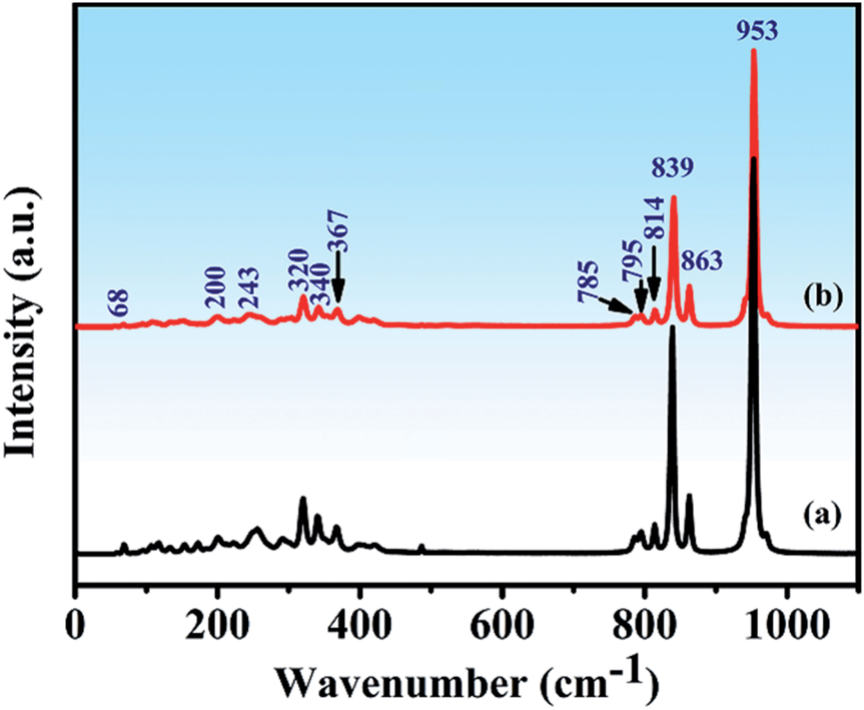
(a) Calculated and (b) Experimental Raman spectra of Y_2_Mo_4_O_15_ crystal.

The Raman spectra and assignment of the lattice modes of Y_2_Mo_4_O_15_ have never been reported in previous studies. From space group theoretical considerations, the primitive cell of the *P*2_1_/*c* structure of Y_2_Mo_4_O_15_ comprises two formula units and 24 atoms, and it can be described in terms of 72 vibrational degrees of freedom. Therefore, the total Brillouin zone centre vibrational modes can be classified according to the irreducible representation of the *C*_2h_ point group of this material as *Γ*_total_ = *Γ*_acoustic_ + *Γ*_optic_ = 30A_g_ + 33A_u_ + 30B_g_ + 33B_u_. The A_g_ and B_g_ modes are Raman active, and A_u_ and B_u_ modes are IR active. It should be remembered, however, that three of these translational motions, A_u_ + 2B_u_, correspond to acoustic modes. These modes involve stretching and bending vibrations of the Mo–O bonds as well as librations of the Y–O polyhedral. The spontaneous Raman spectra of Y_2_Mo_4_O_15_ is shown in Fig. 8 (line b). As observed, twelve key Raman shifts are seen, which are 953 cm^−1^, 863 cm^−1^, 839 cm^−1^, 814 cm^−1^, 795 cm^−1^, 785 cm^−1^, 367 cm^−1^, 340 cm^−1^, 320 cm^−1^, 243 cm^−1^, 200 cm^−1^, and 68 cm^−1^. Most of the calculated frequencies in the calculated Raman spectrum (Fig. 8 (line a)) coincide well with the experimental spectra except for some weak peaks, such as 154 cm^−1^, 172 cm^−1^, 256 cm^−1^, and 292 cm^−1^ peaks. Particularly, the strongest Raman frequency shift is found to be at 953 cm^−1^, which is assigned to the B_g_ mode of asymmetric stretching vibration of the Mo–O bonds by the V_Sim software.

### Phonon dispersion relations

**Fig. 9:**
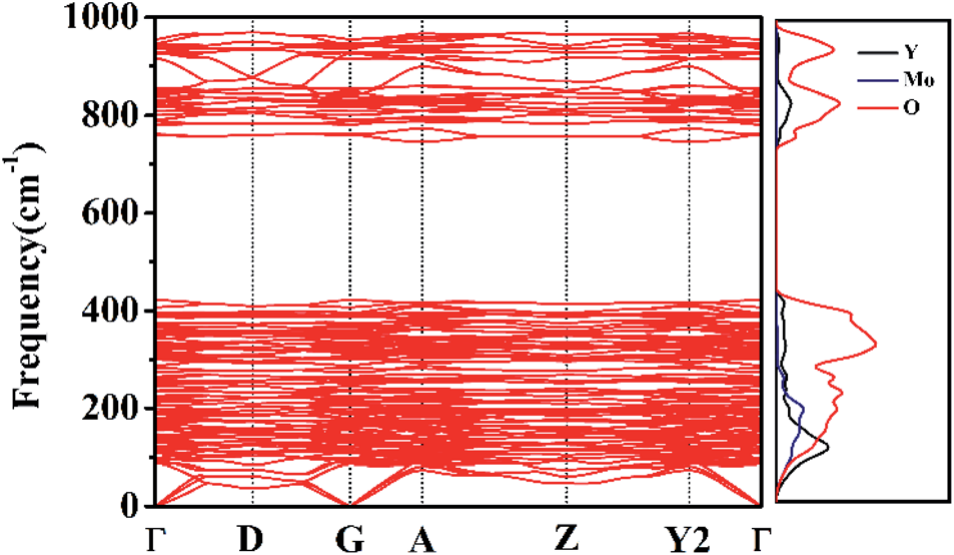
Phonon dispersion curves and phonon density of states of Y_2_Mo_4_O_15_.

Phonon dispersion calculations were performed by sampling other *k*-points in the first Brillouin Zone other than Γ (*k* = 0) using a direct-space approach.^39,40^ Phonon dispersion curves of the monoclinic system in the path Γ→D→G→A→Z→Y2→Γ are reported in Fig. 9 for the 0–1000 cm^−1^ range, alongside the calculated total and atom-projected phonon density of states. From Fig. 9 (right), we can see the asymmetric (953 cm^−1^) and symmetric (863–785 cm^−1^) stretching regions. The total phonon DOS originates mainly from the Mo–O vibrations in accordance with the assignments for Y_2_Mo_4_O_15_.^41^ The spectral region between 367 cm^−1^ and 256 cm^−1^ corresponds to the bending motions. Y, Mo, and O atoms all take part in the vibrations in spite of the motions of Y–O being relatively small. The low frequency region (*ν* < 200 cm^−1^) is characterized by rotational motions. YO_7_ polyhedron and MO_4_ tetrahedron all contribute considerably to the total phonon DOS. They can together affect the Raman behaviour.

## Conclusions

In summary, a new Raman crystal Y_2_Mo_4_O_15_ was grown using the TSSG method. The Y_2_Mo_4_O_15_ crystallizes in the monoclinic space group *P*2_1_/*c* (No. 14) with Mo_4_O_15_ and YO_7_ polyhedra as the basic structural units. The experiments and theoretical calculations indicate that the Y_2_Mo_4_O_15_ crystal has the strongest Raman shift at 953 cm^−1^ caused by the asymmetric stretching vibration of the Mo–O bonds. Moreover, the crystal shows a wide transmission range from 345 nm to 5575 nm. Both the spontaneous Raman spectra and the possibility of rare-earth doping indicate that the Y_2_Mo_4_O_15_ crystal will be a promising Raman and self-Raman crystal.

## Conflicts of interest

There are no conflicts to declare.

## Supplementary Material

RA-011-D0RA08609F-s001

RA-011-D0RA08609F-s002
