# Supplementary material for: Design, growth, and characterization of Y2Mo4O15 crystals for Raman laser applications
Source: RSC Adv. 2021 Jan 4;11(2):1164–71. doi: 10.1039/d0ra08609f (PMC8693878; doi:10.1039/d0ra08609f)
Supplement: RA-011-D0RA08609F-s001 [file RA-011-D0RA08609F-s001.pdf]

Electronic Supplementary Material (ESI) for RSC Advances.
This journal is © The Royal Society of Chemistry 2020

Supporting Information

Design, Growth and Characterization of $Y_2Mo_4O_{15}$ Crystal for Raman laser applications

Xiangmei Wang,[a] Zeliang Gao,*[a] Chunyan Wang,[c] Xiaojie Guo,[a] Youxuan Sun,[a] Yu Jia[b] and Xutang Tao*[a]

a State Key Laboratory of Crystal Materials, Shandong University, Jinan, 250100, China

b Key Laboratory for Special Functional Materials of Ministry of Education, and School of Materials Science and Engineering, Henan University, Kaifeng, Henan, 475001, China

c International Laboratory for Quantum Functional Materials of Henan, and School of Physics and Microelectronics, Zhengzhou University, Zhengzhou, 450001, China

Corresponding Authors*: txt@sdu.edu.cn (X. Tao); gaozeliang@sdu.edu.cn (Z. Gao)

CONTENTS

Table S1. Crystal Data and Structure Refinement for $Y_2Mo_4O_{15}$
Table S2. Selected bond lengths (Å) and angles (deg.) in $Y_2Mo_4O_{15}$
Table S3. Atomic displacement parameters (Å^2) for $Y_2Mo_4O_{15}$

Table S1. Crystal Data and Structure Refinement for $Y_2Mo_4O_{15}$

Empirical formula	$Y_2Mo_4O_{15}$
Formula weight	801.58
Temperature	293 K
Wavelength	0.71073 Å
Crystal system	Monoclinic
Space group	*P2₁/c* (No. 14)
Unit cell dimensions	a = 6.8110(4) Å, α = 90.00°<br>b = 9.5833(5) Å, β = 105.512(7)°<br>c = 10.5124(6) Å, γ = 90°
Volume	661.17(8) $Å^3$
Z	2
Density (calculated)	4.026 g/cm^3
Absorption coefficient	12.429 mm^{-1}
F(000)	732
Crystal size	0.02 × 0.04 × 0.05 mm^3
θ range for data collection	2.903 to 30.323°
Index ranges	$-8 \leq h \leq 8$,<br>$-11 \leq k \leq 10$,<br>$-13 \leq l \leq 13$
Reflections collected	5250
R_{int}	0.1013
Data / restraints / parameters	1342 / 0 / 98
Goodness-of-fit	1.076
Final R indices [>2σ(I)]	R_{obs} = 0.0531, wR_{obs} = 0.1280
R indices [all data]	R_{all} = 0.0563, wR_{all} = 0.1306
Extinction coefficient	0.257(11)
Largest diff. peak and hole	2.188 and -2.120 e.$Å^{-3}$

$R = \Sigma||F_o|-|F_c|| / \Sigma|F_o|$, $wR = \{\Sigma[w(|F_o|^2 - |F_c|^2)^2] / \Sigma[w(|F_o|^4)]\}^{1/2}$ and calc $w=1/[\sigma^2(Fo^2)+(0.0503P)^2+0.0000P]$ where $P=(Fo^2+2Fc^2)/3$

Table S2. Selected bond lengths (Å) and angles (deg.) in $Y_2Mo_4O_{15}$

Mo1—O2	178.3(7)	Mo1—O6	1.735(6)
Mo1—O3	1.746(6)	Y1—O2^{iii}	2.316(6)
Mo1—O7	1.763(7)	Y1—O3^{iv}	2.268(6)
Mo1—O8	1.750(6)	Y1—O4^{v}	2.342(7)
Mo2—Y1^{i}	3.5377(11)	Y1—O5	2.301(7)
Mo2—O1	1.8665(7)	Y1—O6^{vi}	2.355(7)
Mo2—O2^{ii}	2.508(7)	Y1—O7	2.260(6)
Mo2—O4	1.735(6)	Y1—O8^{vi}	2.247(6)
Mo2—O5	1.737(7)	O4^{v}—Y1—O6^{vi}	142.5(2)
O3—Mo1—O2	110.6(3)	O5—Y1—Mo2^{v}	90.51(17)
O3—Mo1—O7	109.1(3)	O5—Y1—O2^{iii}	131.6(2)
O3—Mo1—O8	110.0(3)	O5—Y1—O4^{v}	74.6(2)
O7—Mo1—O2	110.4(3)	O5—Y1—O6^{vi}	141.0(2)
O8—Mo1—O2	109.0(3)	O6^{vi}—Y1—Mo2^{v}	120.83(15)
O8—Mo1—O7	107.7(3)	O7—Y1—Mo2^{v}	89.21(18)
O1—Mo2—Y1^{i}	87.35(3)	O7—Y1—O2^{iii}	83.0(2)
O1—Mo2—O2	75.14(16)	O7—Y1—O3^{iv}	96.1(3)
O2^{ii}—Mo2—Y1	40.75(14)	O7—Y1—O4^{v}	104.4(2)
O4—Mo2—Y1^{i}	35.1(2)	O7—Y1—O5	77.5(3)
O4—Mo2—O1	113.1(2)	O7—Y1—O6	80.1(3)
O4—Mo2—O2^{ii}	73.1(3)	O8^{vi}—Y1—Mo2^{v}	96.83(16)
O4—Mo2—O5	114.1(3)	O8^{vi}—Y1—O2^{iii}	90.1(2)
O4—Mo2—O6	105.1(3)	O8^{vi}—Y1—O3^{iv}	82.3(2)
O5—Mo2—Y1^{i}	108.7(2)	O8^{vi}—Y1—O4^{v}	87.2(2)
O5—Mo2—O1	115.6(2)	O8^{vi}—Y1—O5	118.1(2)
O5—Mo2—O2^{ii}	79.3(3)	O8^{vi}—Y1—O6^{vi}	83.2(2)
O6—Mo2—Y1^{i}	136.7(2)	O8^{vi}—Y1—O7	163.1(3)
O6—Mo2—O1	103.5(2)	Mo2^{vii}—O1—Mo2	180
O6—Mo2—O2^{ii}	176.8(3)	Mo1—O1—Mo2^{viii}	130.0(3)
O6—Mo2—O5	103.9(3)	Mo4—O2—Y1^{iii}	135.6(4)
O2^{iii}—Y1—Mo2^{v}	44.99(18)	Y1^{iii}—O2—Mo2^{viii}	94.3(2)
O2^{iii}—Y1—O4^{v}	68.0(2)	Mo1—O3—Y1$^{i\ x}$	164.7(4)
O2^{iii}—Y1—O6^{vi}	75.9(2)	Mo2—O4—Y1^{i}	119.7(3)
O3—Y1—Mo2^{v}	163.98(18)	Mo2—O5—Y1	143.6(4)
O3—Y1—O2^{iii}	150.6(3)	Mo2—O6—Y1^{x}	163.5(4)
O3—Y1—O4	139.3(2)	Mo1—O7—Y1	147.8(4)
O3^{iv}—Y1—O5	76.0(2)	Mo1—O8—Y1^{x}	144.9(4)
O3^{iv}—Y1—O6	75.1(2)		
O4—Y1—Mo2^{v}	25.21(16)		

(i) 2-x, -0.5+y, 1.5-z; (ii) 1+x, 1.5-y, 0.5+z; (iii) 1-x, 2-y, 1-z; (iv) x, 1.5-y, 0.5+z;
(v) 2-x, 0.5+y, 1.5-z; (vi) 1-x, 0.5+y, 1.5-z; (vii) 2-x, 1-y, 2-z; (viii) -1+x, 1.5-y, -0.5+z;
(ix) x, 1.5-y, -0.5+z; (x) 1-x, -0.5+y, 1.5-z.

Table S3. Atomic displacement parameters (Å^2) for $Y_2Mo_4O_{15}$

Atom	U^{11}	U^{22}	U^{33}	U^{12}	U^{13}	U^{23}
Mo1	0.0125(5)	0.0143(5)	0.0097(5)	0.0002(2)	0.0032(4)	0.0013(2)
Mo2	0.0104(5)	0.0150(5)	0.0117(5)	0.0004(2)	0.0012(4)	0.0008(3)
Y1	0.0112(6)	0.0135(5)	0.0106(5)	0.0005(3)	0.0031(4)	0.0002(3)
O1	0.019(6)	0.029(5)	0.022(5)	0.014(4)	-0.003(4)	-0.001(4)
O2	0.019(4)	0.019(3)	0.021(3)	0.006(2)	0.008(3)	-0.002(3)
O3	0.023(4)	0.023(3)	0.020(3)	-0.006(2)	0.010(3)	0.001(3)
O4	0.010(3)	0.031(4)	0.017(3)	-0.010(3)	0.005(3)	-0.001(3)
O5	0.015(4)	0.021(3)	0.024(3)	0.004(2)	0.003(3)	0.005(2)
O6	0.008(3)	0.032(4)	0.022(3)	-0.006(3)	0.008(3)	0.000(3)
O7	0.023(4)	0.026(4)	0.016(3)	-0.008(3)	0.002(3)	-0.002(3)
O8	0.018(4)	0.028(3)	0.019(3)	0.011(3)	0.010(3)	0.011(3)
